# Comparative analysis of organellar genomes between diploid and tetraploid *Chrysanthemum indicum* with its relatives

**DOI:** 10.3389/fpls.2023.1228551

**Published:** 2023-08-18

**Authors:** Huihui Liu, Zhuangwei Hou, Lei Xu, Qing Ma, Min Wei, Luke R. Tembrock, Shuo Zhang, Zhiqiang Wu

**Affiliations:** ^1^ China Resources Sanjiu Medical & Pharmaceutical Co., Ltd, Shenzhen, China; ^2^ Shenzhen Branch, Guangdong Laboratory for Lingnan Modern Agriculture, Genome Analysis Laboratory of the Ministry of Agriculture, Agricultural Genomics Institute at Shenzhen, Chinese Academy of Agricultural Sciences, Shenzhen, Guangdong, China; ^3^ Department of Agricultural Biology, Colorado State University, Fort Collins, CO, United States; ^4^ National Key Laboratory of Crop Genetic Improvement, Huazhong Agricultural University, Wuhan, Hubei, China; ^5^ Kunpeng Institute of Modern Agriculture at Foshan, Foshan, China

**Keywords:** *Chrysanthemum indicum*, Asteraceae, organelle genome, intragenomic gene transfers, phylogenetic analysis, tetraploid, variants

## Abstract

*Chrysanthemum indicum*, a species native to Eastern Asia is well known as one of the progenitor species of the cultivated *Chrysanthemum* which is grown for its ornamental and medicinal value. Previous genomic studies on *Chrysanthemum* have largely ignored the dynamics of plastid genome (plastome) and mitochondria genome (mitogenome) evolution when analyzing this plant lineage. In this study, we sequenced and assembled the plastomes and mitogenomes of diploid and tetraploid *C. indicum* as well as the morphologically divergent variety *C. indicum* var. *aromaticum*. We used published data from 27 species with both plastome and mitogenome complete sequences to explore differences in sequence evolution between the organellar genomes. The size and structure of organellar genome between diploid and tetraploid *C. indicum* were generally similar but the tetraploid *C. indicum* and *C. indicum* var. *aromaticum* were found to contain unique sequences in the mitogenomes which also contained previously undescribed open reading frames (ORFs). Across *Chrysanthemum* mitogenome structure varied greatly but sequences transferred from plastomes in to the mitogenomes were conserved. Finally, differences observed between mitogenome and plastome gene trees may be the result of the difference in the rate of sequence evolution between genes in these two genomes. In total the findings presented here greatly expand the resources for studying *Chrysanthemum* organellar genome evolution with possible applications to conservation, breeding, and gene banking in the future.

## Background

*Chrysanthemum indicum*, a perennial herbaceous plant in the Asteraceae family, is widely used as a medicinal, ornamental, and food plant (Luo and Li, 2013). Previous studies have found that *C. indicum* possesses variable traits across the species range and also varies in ploidy level (Li et al., 2013; Klie et al., 2014; Qi et al., 2021), including diploid, tetraploid, and hexaploid (Si-Lan. et al., 1998; C. et al., 2004; El-Twab and Kondo, 2007; Li et al., 2013; Klie et al., 2014). For example *C. indicum* from coastal saline soils were noted to be dwarfed, while those found on Mount Lu (Jiangxi Province, China) were found to have a greater density of trichomes on their leaves than plants from the other regions (Luo and Li, 2013). Frequent gene flow has been reported between species in the *Chrysanthemum* genus (Si-Lan. et al., 1998; C. and S, 2002; C. et al., 2004; El-Twab and Kondo, 2007; Luo et al., 2016), as well as among different accessions within *C. indicum* (Li et al., 2013; Luo et al., 2016; Ma et al., 2020; Qi et al., 2021). In previous phylogenetic studies about *Chrysanthemum* genus, *C. indicum* were frequently found to be most closely related with *C. zawadskii* and C*. nankingenese* (C. et al., 2004). However, substantial gene flow has led to conflicting phylogenetic outcomes within the genus and among wild populations of *C. indicum* (C. et al., 2004; El-Twab and Kondo, 2007). Previous studies have suggested that tetraploid *C. indicum* originated from hybridization between diploid *C. indicum* and related species (Li et al., 2013), while other studies have found that some *C. indicum* polyploid populations were homoeologous polyploids (Li et al., 2014). Within *C. indicum* the variety *C. indicum* var. *aromaticum* was described to account for individuals that possess thicker leaf blades and glandular hairs (Luo and Li, 2013), but studies on this variety have also found that gene flow has occurred between it and other *C. indicum* lineages (Luo and Li, 2013; Yuan et al., 2022). Much of the research regarding *C. indicum* has been in its contribution to the Chinese cultivated *Chrysanthemum* which is an allohexaploid lineage used as a horticultural ornament (Si-Lan. et al., 1998; C. and S, 2002; C. et al., 2004; El-Twab and Kondo, 2007). The above-mentioned evidence for genetic admixture in *Chrysanthemum* has been derived from the nuclear loci with little known about how mitogenomes and plastomes have been sorted and evolved during episodes of admixture and increases in ploidy.

Sequence data from plant mitogenomes and plastomes has been widely used for plant phylogenetic research due to the presence of conserved nonrecombinant sequences that flank more rapidly evolving regions (Kelchner, 2002; Rogalski et al., 2015; Uncu et al., 2015). For example, the phylogeny of the three large families: Arecaceae, Moraceae, and Saxifragaceae, have been resolved with plastome data (Liu et al., 2020; Zhang et al., 2022; Yao et al., 2023). With the decreasing cost of high-throughput sequencing and the improvement of assembly techniques, an increasing number of studies are using complete organellar genomes instead of one or several loci to conduct phylogenetic and evolutionary studies in plants. Currently, 1852 plastomes (mostly from different varieties of the same species) and 62 mitogenomes of Asteraceae are available in GenBank. The smaller number of published mitogenomes is a result of the difficulty in assembling plant mitochondrial genomes (high frequency of rearrangements) using short read sequencing techniques (Uncu et al., 2015). For example, it is difficult to resolve complex mitogenome assembly results with different contig-repeat-contig combinations by using only short read sequencing data (Zhang et al., 2023). However, the issue of complete mitogenome assembly is gradually being addressed with the use of long-read sequencing methods. Although many organellar genomes have been published from the Asteraceae, most studies have been limited to phylogenetic research, without further exploring the evolutionary patterns within a species or how organellar genome evolution correlates with changes in nuclear genome ploidy (Xia et al., 2016; Kim et al., 2018; Won et al., 2018; Feng et al., 2020; Xia et al., 2021a; Xia et al., 2021b; Yu et al., 2021; Masuda et al., 2022).

In this study, we sequenced and assembled the complete mitogenomes and plastomes of diploid and tetraploid *C. indicum*, as well as *C. indicum* var. *aromaticum*, and compared them with those of 23 Asteraceae species for which public data was available for both organellar genomes. This is the first study to explore the evolutionary histories of varieties of *C. indicum* with different ploidy levels and phenotypic variations using organellar genomes as a basis for historical inference. We compared the differences of the sequence and structure of organellar genomes among different varieties of *C. indicum*, resolved phylogenetic relationships among different varieties of *C. indicum*, analyzed the diversity of simple sequence repeats (SSRs), and quantified the gene transfers from plastomes to mitogenomes.

## Results

### Genome characteristics of *Chrysanthemum* organelle

**Figure 1:**
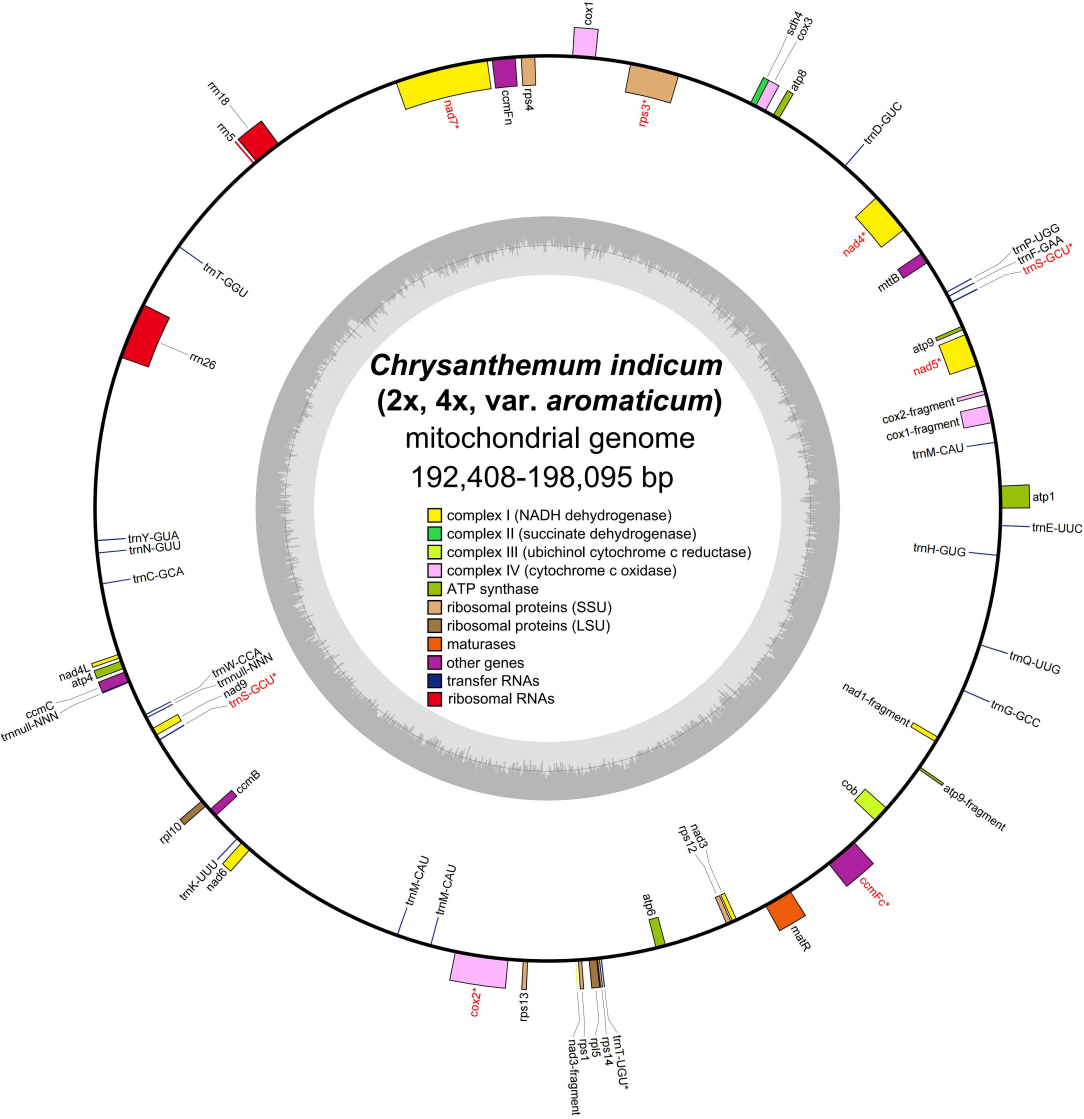
Mitogenome structure from three *C. indicum* accessions in this study. * indicates gene containing intron (s).

By utilizing long PacBio HiFi reads, the mitogenomes of diploid (2x) and tetraploid (4x) *C. indicum* were assembled with published *C. indicum* mitogenome (NCBI accession number: MH716014.1) used to extract sequences from whole genome data. In addition the mitogenome of *C. indicum* var. *aromaticum* was also assembled with Illumina reads. The mitogenome of *C. indicum* (2x) was assembled into a single circular genome, and was used to resolve the mitogenome of *C. indicum* (4x) and *C. indicum* var. *aromaticum* with multiple linkage structures (**Figure S1**). The sizes of the three circular mitogenome were 192,408 bp with 45.51% GC content for *C. indicum* (2x), 193,563 bp with 45.43% GC content for *C. indicum* (4x), and 198,095 bp with 45.33% GC content for *C. indicum* var. *aromaticum*, similar with that in found in most Asteraceae species sequenced at present (**Figure 1**). All three of these newly assembled mitogenomes contain 51 unique genes, including 33 protein coding genes (PCGs) genes, three rRNA genes, and 16 tRNA, with *trnS-GCU* having two copies and *trnM-CAU* having three copies in *C. indicum* (2x) and *C. indicum* (4x), but five copies in *C. indicum* var. *aromaticum* (**Table 1**). Among the PCGs, eight genes contained introns, three of which (*ccmFC*, *nad4* and *rps3*) contained one intron, and five others (*cox2*, *nad1*, *nad2*, *nad5*, and *nad7*) contained two or more introns. Among the three mitogenomes, all PCGs are single copy, except *atp9* that has two copies in *C. indicum* var. *aromaticum*.

**Table 1:** Gene composition in the mitogenome of *Chrysanthemum indicum*.

Group of genes		Name of genes
Core genes	ATP synthase	*atp1, atp4, atp6, atp8, atp9*
	Cytochrome c biogenesis	*ccmB, ccmC, ccmFc, ccmFn*
	Ubichinol cytochrome c reductase	*cob*
	Cytochrome c oxidase	*cox1, cox2, cox3*
	Maturases	*matR*
	Transport membrane protein	*mttB*
	NADH dehydrogenase	*nad1, nad2, nad3, nad4, nad4L, nad5, nad6, nad7, nad9*
Variable genes	Large subunit of ribosome	*rpl5, rpl10*
	Small subunit of ribosome	*rps1, rps3, rps4, rps12, rps13, rps14*
	Succinate dehydrogenase	*sdh4*
rRNA genes	Ribosomal RNAs	*rrn5, rrn18, rrn26*
tRNA genes	Transfer RNAs	*trnY-GUA, trnW-CCA, trnT-GGU,trnT-UGU,trnS-GCU (×2), trnQ-UUG, trnP-UGG*, *trnN-GUU, trnM-CAU (×3, ×5)*, *trnK-UUU, trnH-GUG, trnG-GCC, trnF-GAA, trnE-UUC, trnD-GUC, trnC-GCA*
Plastid-derived	partial	*psaB, psaA, ycf2, rps11, 16S rRNA*
Plastid-derived	complete	*petL, petG, trnW-CCA, trnW-CCA,trnN-GUU,trnH-GUG,trnM-CAU,trnP-UGG*

All three newly assembled chloroplast genomes maintain the classical dumbbell-shaped genome structure. The plastome sizes of the three samples were very similar at 151,033 bp for *C. indicum* (2x), 151,036 bp for *C. indicum* (4x) and 151,053 bp for *C. indicum* var. *aromaticum* with the same GC content at 37.47% for all these plastomes (**Figure S2**). All three of these newly assembled plastomes of *Chrysanthemum* contain 109 unique genes, including 80 PCGs, 25 tRNAs, and four rRNAs with the IR region containing six protein coding genes, eight tRNAs and four rRNAs, with 13 PCGs containing introns, and two of these (*rps12* and *ycf3*) have more than one intron. Overall, compared to mitogenomes, these three plastomes differ only slightly in a few regions and are generally conserved in sequence similarity.

#### Differences between organelle genome sequences of *Chrysanthemum*

**Figure 2:**
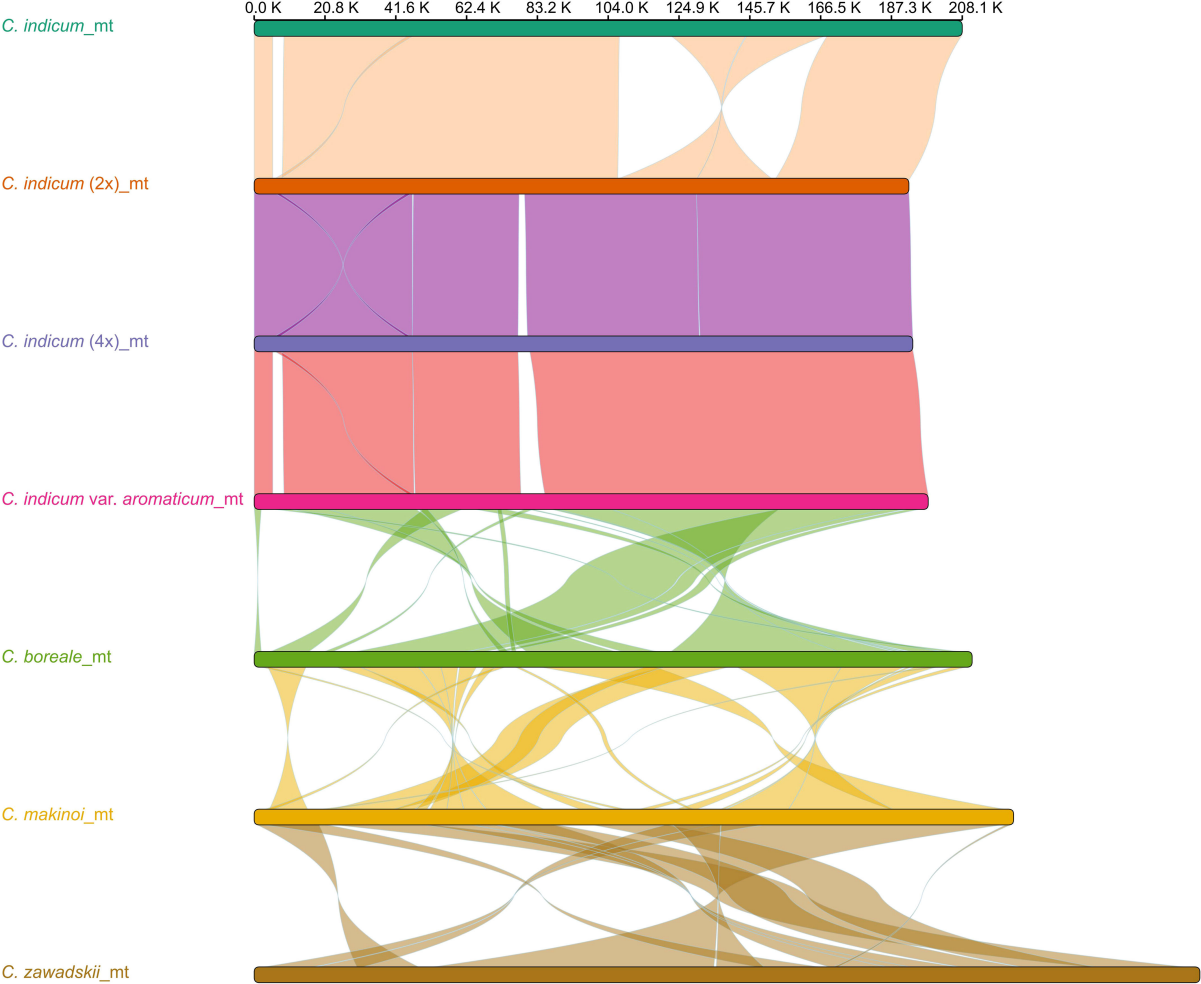
Synteny between *Chrysanthemum* mitogenomes. Mitogenomes from seven accessions with sequence rearrangements longer than 1 Kb between two pairs shown.

To better understand organellar genome evolution in *Chrysanthemum*, the three mitogenomes from *Chrysanthemum indicum* assembled in this study were used for genome-wide collinear comparisons with published mitogenomes of four *Chrysanthemum* species *C. indicum* (MH716014), *C. boreale* (NC_039757), *C. makinoi* (OU343227), and *C. zawadskii* (ON053202) (**Figure 2**). The four accessions from *C. indicum* have a more similar mitogenome structure in the genome-wide alignment results for sequence lengths greater than 1 Kb, except in *C. indicum* (MH716014) which did not have the 22.6 Kb and 23.7 Kb inversions found in the other three species (**Figure 2**). Between the four *Chrysanthemum* species mitogenomes highly divergent rearrangements were found pointing to a process of rapid structural turnover between species (**Figure 2**). Among the three newly assembled mitogenomes, *C. indicum* (4x) has a unique sequence of 2.8 Kb compared to *C. indicum* (2x), on which two ORFs encoding proteins of 496aa and 72aa in length were predicted. In *C. indicum* var. *aromaticum* two unique sequences of length 3.3 Kb and 5.6 Kb were found with ORFs encoding proteins of 307aa, 165aa, and 73aa in length predicted on the 3.3 Kb sequence. On the 5.6 Kb sequence ORFs encoding proteins 383 aa, 346 aa, 94 aa, 72 aa, and 71 aa in length, and two t*rnM-CAU* were predicted (**Table S2**). Comparative sequence analysis of the plastomes corresponding to seven *Chrysanthemum* accessions showed that the plastome sequences were conserved within the genus (**Figure S3**). In addition, to understand the structural differences of mitogenomes within cultivated *C. indicum*, the three newly assembled mitogenomes were compared separately for intra-genomic collinearity, and it was found that these mitogenomes all contain few repetitive sequences, which explains the relatively small size of these mitogenomes. The *C. indicum* (2x) and *C. indicum* (4x) mitogenomes were more similar in intra-genomic collinearity patterns and showed similarity between their genome structure (**Figure S4**). In general, the mitogenomes showed large structural differences between species within *Chrysanthemum* and greater synteny within species while the plastomes were structurally conserved across all samples.

**Figure 3:**
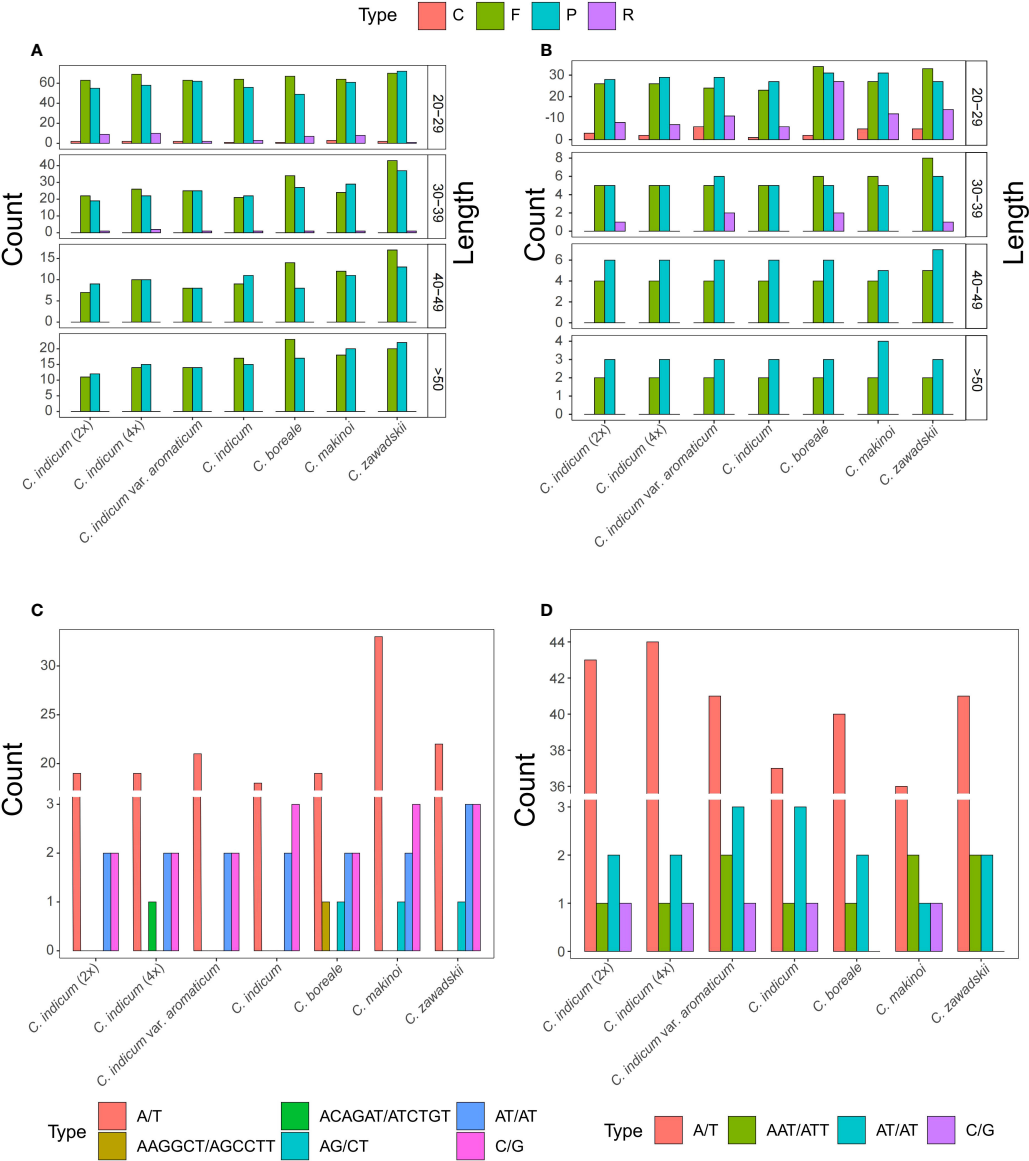
Repeat sequences in *Chrysanthemum* organellar genomes. **(A)** Mitogenome repeats identified with REPuter include F (forward), R (reversed), C (complement) and P (palindromic); **(B)** Plastome repeats identified with REPuter include F, P, C, and R repeats; **(C)** The SSRs identified from *Chrysanthemum* mitogenomes; **(D)** The SSRs identified from *Chrysanthemum* plastomes.

To further understand the differences between organellar genomes of *Chrysanthemum* repeat sequences were annotated and compared among seven *Chrysanthemum* accessions (**Figure 3**). The *Chrysanthemum* organellar genomes contained repeat types Complement (C), Forward (F), Palindromic (P), and Reverse (R) at different ratios, with repeats less than 50 bp being the most common (**Figures 3A, B**). In all accessions, the number of repeats of the F and P type were dominant in both genomes but in plastomes the repeat types were more evenly distributed especially among the shorter repeat sequences (**Figures 3A, B**). The abundance of F and P type repeats in the mitogenome is correlated with larger mitogenomes. To better understand the dynamics of repetitive sequences in the organelles of the *Chrysanthemum* plastome and mitogenome, SSR sequences of seven accessions were analyzed (**Figures 3C, D**). A total of 23, 24, 25, 23, 25, 39, and 29 SSRs were detected in the mitogenomes of *C. indicum* (2x), *C. indicum* (4x), *C. indicum* var. *aromaticum*, *C. indicum*, *C. boreale*, *C. makinoi*, and *C. zawadskii* (**Figure 3C**), respectively, and a total of 47, 48, 47, 42, 43, 40, and 45 SSRs were detected in the plastomes of *C. indicum* (2x), *C. indicum* (4x), *C. indicum* var. *aromaticum*, *C. indicum*, *C. boreale*, *C. makinoi*, and *C. zawadskii* (**Figure 3D**), respectively. The number of SSRs in plastomes was greater than that in the corresponding mitogenomes (**Figures 3C, D**). The number of A/T SSRs in the plastomes and mitogenomes far exceeded the combined number of all other types of SSRs while the mitogenome of *C. makinoi* had a much higher number of A/T repeats than the other accessions (**Figures 3C, D**). The mitogenomes of all four accessions from *C. indicum* did not contain AG/CT SSRs, but *C. indicum* (4x) possessed a unique ACAGAT/ATCTGT SSR, and *C. boreale* possessed a unique AAGGCT/AGCCTT SSR (**Figure 3C**). In the plastomes the number and type of SSRs are more conserved, except for *C. boreale* and *C. zawadskii* that did not contain any C/G SSRs, all other accessions contained four C/G SSR loci (**Figure 3D**).

### Intracellular gene transfer between organellar genomes

**Figure 4:**
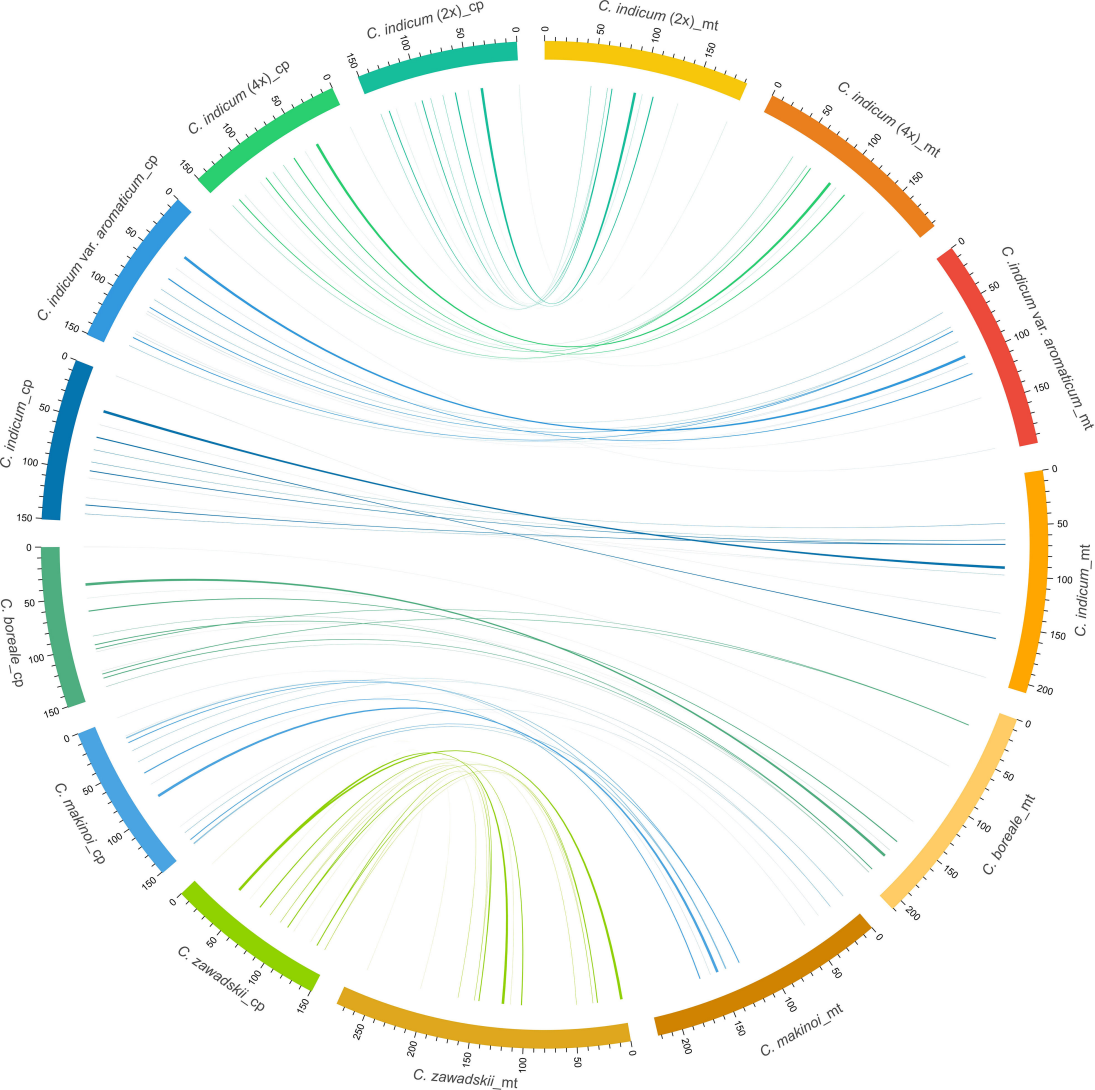
Schematic diagram of homologous sequences identified among the two organellar genomes of seven *C. indicum* accessions. Plastomes are on the left and corresponding mitogenomes are on the right. Lengths are given in Kb.

In plants, the exchange of genetic material between cellular compartments (nucleus, mitochondria, and chloroplasts) is often referred to as Intracellular Gene Transfer (IGT) (Bergthorsson et al., 2003; Aubin et al., 2021). Characterizing these IGT events is key to understanding the genetics and evolution of organellar genomes. We did this in *Chrysanthemum*, by quantifying the sequence transfers from plastomes to mitogenomes (**Figure 4**). Each accession had a total of 8-10 (from 5220-9176 bp in total length) fragments from a plastomic origin integrated into 8-12 (5212-9164 bp) locations in the mitogenome. The least number (8) of transferred sequences was in *C. indicum* (2x) and the most (10) in *C. makinoi*. The length of the transferred fragments ranged from the shortest at 79 bp to the longest of 2556 bp (**Table 2**; **Table S1**). Correlation analyses indicated that the number of plastome transferred sequences and the size of the corresponding mitogenomes were positively correlated, as the number and length of plastome transfer sequences was greater for accessions with large mitogenomes (**Figure S5**). The transferred sequences across the seven accessions were conserved in respect to both size and sequence of origin. Specifically all accessions contained seven completely transferred gene sequences (*petL*, *petG*, *trnM-CAU*, *trnH-GUG*, *trnN-GUU*, *trnW-CCA*, and *trnP-UGG*) and five partially transferred gene sequences (*16S rRNA*, *rps11*, *ycf2*, *psaB*, and *psaA*) (**Table 2**). Interestingly, *C. indicum* var. *aromaticum*, *C. boreale*, and *C. makinoi* have partially transferred sequences of the *23S rRNA* gene, but the lengths of these fragments vary from 135 bp in *C. indicum* var. *aromaticum* to 357 bp and 443 bp in *C. makinoi*, and 238 bp in *C. boreale* (**Table S1**). These results suggest that the plastomic fragments were transferred to the mitogenome predating speciation, and that shared transfers specifically between *C. indicum* var. *aromaticum*, and other *Chrysanthemum* species may be the result of past hybridization, incomplete lineage sorting, or retention of ancestral transfers lost in other *C. indicum* species.

**Table 2:** MTPTs (Mitochondrial plastid DNAs) detected in *C. indicum*.

Accession	Length	Plastome		Identity	Mitogeome		Transferred genes
		Start	End		Start	End	
*C. indicum* (2x)	2548	36725	39279	98.122	90427	92974	*psaB* (partial)-*psaA* (partial)
*C. indicum* (2x)	1057	64491	65565	78.437	109975	111031	*petL*-*petG*-*trnW* (CCA)-*trnP* (UGG)
*C. indicum* (2x)	247	143167	143422	93.750	63521	63767	*ycf2* (partial)
*C. indicum* (2x)	247	90424	90679	93.750	63521	63767	
*C. indicum* (2x)	266	77775	78040	89.098	46826	47091	*rps11* (partial)
*C. indicum* (2x)	859	133864	134727	73.761	67820	68678	*16S rRNA* (partial)
*C. indicum* (2x)	859	99119	99982	73.761	67820	68678	
*C. indicum* (2x)	84	126955	127037	96.429	99185	99268	*trnN-GUU*
*C. indicum* (2x)	84	106809	106891	96.429	99185	99268	
*C. indicum* (2x)	80	9	88	97.500	189184	189263	*trnH-GUG*
*C. indicum* (2x)	79	51936	52014	97.468	136251	136329	*trnM-CAU*
*C. indicum* (4x)	2548	36733	39287	98.161	91314	93888	*psaB* (partial)-p*saA* (partial)
*C. indicum* (4x)	1057	64493	65567	78.527	110889	111945	*petL*-*petG*-*trnW* (CCA)-*trnP* (UGG)
*C. indicum* (4x)	247	143170	143425	93.359	63195	63441	*ycf2* (partial)
*C. indicum* (4x)	247	90426	90681	93.359	63195	63441	
*C. indicum* (4x)	266	77777	78042	89.098	46500	46765	*rps11* (partial)
*C. indicum* (4x)	859	133867	134730	73.761	67494	68352	*16S rRNA* (partial)
*C. indicum* (4x)	859	99121	99984	73.761	67494	68352	
*C. indicum* (4x)	84	126958	127040	96.429	100099	100182	*trnN-GUU*
*C. indicum* (4x)	84	106811	106893	96.429	100099	100182	
*C. indicum* (4x)	80	9	88	97.500	190339	190418	*trnH-GUG*
*C. indicum* var. *aromaticum*	2556	36703	39257	98.905	95892	98447	*psaB* (partial)-*psaA* (partial)
*C. indicum* var. *aromaticum*	1057	64470	65544	78.437	115448	116504	*petL*-*petG*-*trnW* (CCA)-*trnP* (UGG)
*C. indicum* var. *aromaticum*	247	143187	143442	93.750	64063	64309	*ycf2* (partial)
*C. indicum* var. *aromaticum*	247	90434	90689	93.750	64063	64309	
*C. indicum* var. *aromaticum*	266	77788	78053	89.098	47356	47621	*rps11* (partial)
*C. indicum* var. *aromaticum*	859	133884	134747	73.761	68362	69220	*16S rRNA* (partial)
*C. indicum* var. *aromaticum*	859	99129	99992	73.761	68362	69220	
*C. indicum* var. *aromaticum*	135	129724	129860	90.511	80683	80817	*23S rRNA* (partial)
*C. indicum* var. *aromaticum*	135	104016	104152	90.511	80683	80817	
*C. indicum* var. *aromaticum*	84	126968	127050	96.429	104657	104740	*trnN-GUU*
*C. indicum* var. *aromaticum*	84	106826	106908	96.429	104657	104740	
*C. indicum* var. *aromaticum*	80	9	88	97.500	194870	194949	*trnH-GUG*
*C. indicum* var. *aromaticum*	79	51917	51995	97.468	141935	142013	*trnM-CAU*

### Phylogenetic relationships

**Figure 5:**
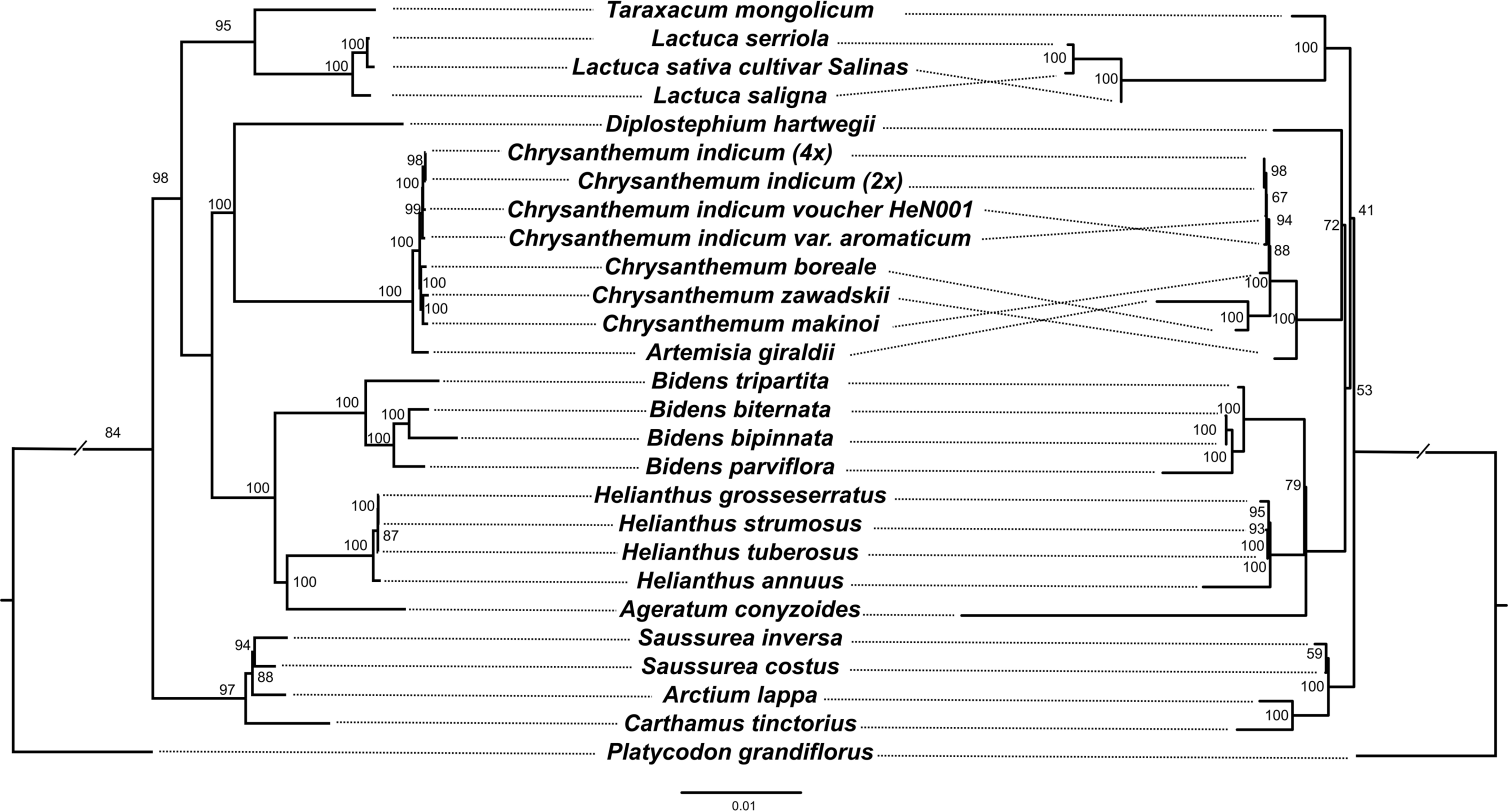
Comparison of ML trees between different gene sets. Left. ML tree based on 80 plastid genes. Right. ML tree based on 32 mitochondrial genes. Numbers at nodes are bootstrap values.

A maximum likelihood (ML) phylogenetic tree of 26 Asteraceae species (*Platycodon grandifiorus* was set as outgroup) with complete mitogenome and plastome data was constructed based on 80 plastome genes and 32 mitogenome genes, respectively (**Figure 5**). In our study, the phylogenetic topology at tribe level was generally consistent with previous research (Zhang et al., 2021). However, the internal phylogenetic relationships within the Anthemideae were somewhat different. On the mitogenome tree, *C. boreale* grouped with *Artemisia giraldii*, while on plastome tree, it grouped with *Chrysanthemum*. This may be due to the fact that the four mitogenome genes (*atp9*, *mttb*, *nad2* and *nad6*) in *C. boreale* show greater similarity with that in *A. giraldii*, especially the *atp9* gene which was identical between the two species. In a previous study, it was suggested that *C. boreale* may be an early diverging species within *Chrysanthemum*, and thus may have retained more ancestral characters (El-Twab and Kondo, 2007). From the phylogenetic tree all four genomes of *C. indicum* clustered together in both plastome and mitogenome analyses. The phylogenetic analysis also suggested that the maternal donor of the tetraploid *C. indicum* may be the diploid *C. indicum*, while *C. indicum* var. *aromaticum* appears to have undergone some degree of divergence from the other *C. indicum*.

With the set model parameters, we obtained two time tree result based on plastome estimation and mitogenome estimation (**Figures S6, S7**). From the results, the 95% intervals obtained for plastomes are more convergent, while those for mitogenomes are broader, which may be related to the mutation rate and the number of mutation sites in mitochondria (**Figure S6**). Here we take the plastome results as the basis. From the results, we learnt that the *C. indicum* was born at 1.43 Mya, and *C. indicum* var. *aromaticum* and *C. indicum* were separated at 0.81 Mya; while tetraploid *C. indicum* and diploid *C. indicum* were separated at 0.38 Mya (**Figure S7**).

## Discussion

### Interspecific diversity and intraspecific conservation of mitogenome structure and sequence in *Chrysanthemum*

Plant mitogenomes have undergone tremendous structural changes during evolution, which have resulted in a near 200-fold variation in mitogenome size among plants (from ~66 Kb of *Viscum scurruloideum* to ~12 Mb of *Larix sibirica*) and frequent rearrangements with low gene density, in contrast to plant plastomes which are conserved in genome size from 115-165 Kb and containing a dumbbell-shaped genome structure (Putintseva et al., 2020; Wu et al., 2020). Owing to the release of a large amount of plant whole genome sequencing data, an increasing number of mitogenomes have been assembled and subjected to comparative studies, in which many mitogenomes have been found to exhibit a reticulated complex genomic structure. The diversity of many mitochondrial genomic structures is thought to be the result of recombination mediated by repetitive sequences (Wu and Sloan, 2019). However, the three mitogenomes of *C. indicum* newly assembled in this study show a simple monocyclic structure, along with a low number and percentage (0.9%-2.2%) of repetitive sequences, which may account for the structure and relatively small size at just over 40 Kb longer than their respective plastomes. In a comparison among four accessions of *C. indicum*, it was found that the mitogenome structure and sequence within species is relatively conserved, in contrast to large differences observed among mitogenomes within cultivated sorghum (Zhang et al., 2023). Meanwhile, only a 2.8 Kb segment of the mitogenome differed between accessions of *C. indicum* of different ploidy levels, two ORFs encoding proteins 496aa and 72aa in length were predicted on this segment, and the functions of these two ORFs have yet to be investigated by mitochondrial gene editing techniques (Kazama et al., 2019). However, when comparing among four different species in *Chrysanthemum*, it was found that the mitogenomes differed not only in size, but also in structure, mainly in genome sequence order. In conclusion, the relationship between mitogenome sequence and structural variation within *Chrysanthemum* and what effect this has on molecular function needs to be further investigated from a greater number of samples. What factors are driving structural conservation within *C. indicum* and divergence between species should also be investigated in greater depth.

### Conserved intracellular gene transfers between organellar genomes in *Chrysanthemum* mitogenomes

IGT occurs frequently and continuously during nucleoplasmic coevolution between different genomes in plant cells (Zhang et al., 2020). In this study, we assembled the mitogenomes and plastomes of three *C. indicum* accessions and examined the sequence transfers from plastome to mitogenome in combination with four published organellar genomes of species in *Chrysanthemum*. It was found that the number and the size of these transferred sequences within *Chrysanthemum* showed a positive correlation with the size of the mitogenome. The transferred genes were conserved among the accessions, and the size of the transferred fragments containing these genes was also generally conserved. All samples contained seven completely transferred gene sequences from the plastome (*petL*, *petG*, *trnM-CAU*, *trnH-GUG*, *trnN-GUU*, *trnW-CCA*, and *trnP-UGG*) and five partially transferred genes (*16S rRNA*, *rps11*, *ycf2*, *psaB*, and *psaA*). These results suggest that these sequence transfers occurred prior to the divergence of *Chrysanthemum* species and have been retained through time albeit in different locations of the genome given rapid turnover in structure from recombination. Interestingly, all four accessions of *C. indicum* have the same transferred gene, except for *C. indicu*m var. *aromaticum* that has an additional 135 bp transferred sequence containing a partially transferred sequence of the *23S rRNA* gene. This transferred fragment shared between *C. indicum* var. *aromaticum* and the other species in *Chrysanthemum* may suggest that this lineage is early diverging or that introgression or incomplete lineage sorting may have been involved in the evolution of this variety.

### Phylogenetic relationships

We observed different topologies between phylogenetic analysis based on plastome and mitogenome sequences. Overall, the results obtained from chloroplasts are consistent with previous studies based on nuclear genes, while mitochondria tend to exhibit conflicting resolutions compared with nuclear gene results in adjacent clades (Zhang et al., 2021). This may be due to the slow mutation rate in sequences that have been observed in plant mitogenomes (Duff and Nickrent, 1999; Xi et al., 2013; Grewe et al., 2014). Additionally, the high frequency of RNA editing sites in mitochondrial genes imposes difficulties for mitochondrial gene tree construction (Drouin et al., 2008; Knoop, 2011). For example, in angiosperms, the number of editing sites in the mitogenome often ranges from 200 to 500, while in the plastome, there are only 30 to 50 editing sites (Malek et al., 1996; Knoop, 2011; Zhang et al., 2021). Furthermore, the editing sites in the mitochondrial genome are not only evident in the coding region, but also in tRNA, introns, and 5’ and 3’ untranslated regions (Malek et al., 1996; Oldenkott et al., 2014). Our study supports the view that mitochondrial genes may not be appropriate for plant phylogenetic inferences at some levels of taxonomic organization (Malek et al., 1996; Drouin et al., 2008; Zhang et al., 2021).

## Conclusions

The current research used highly accurate long-read HiFi sequencing data to assemble mitogenomes from diploid and tetraploid *C. indicum* and short read sequencing data for *C. indicum* var. *aromaticum* using assembly software GSAT. We compared the differences among mitogenomes from different species within *Chrysanthemum* and different accessions of *C. indicum* using the newly assembled and previously published organellar genomes, and found that between species large structural differences are present, but within *C. indicum* species the structure is more conserved between accessions of different ploids. Meanwhile the plastome is structurally conserved across Asteraceae. Transferred genes from plastome to mitogenome showed high levels of sequence conservation within the accessions of different ploids from *C. indicum* species. In addition, we resolved phylogenetic relationships using plastome and mitogenome CDSs of 27 Asteraceae accessions, with *Platycodon grandiflorus* as an outgroup and found conflicting resolutions between these gene sets potentially because of the extremely slow rates of evolution among mitogenome CDSs.

## Methods

### Samples

The samples collected for this study were all from the *Chrysanthemum indicum* germplasm base of China Resources Sanjiu Medical & Pharmaceutical Co., Ltd in Yangxin County, Huangshi City, Hubei Province. The total genomic DNA was extracted using a Cetyltrimethylammonium Bromide (CTAB) method (Arseneau et al., 2017). The same DNA sample was used for Illumina sequencing and HiFi sequencing by using a Hiseq Xten PE150 sequencing platform (Emerman et al., 2017) and PacBio Sequel II sequencing platform respectively (Eid et al., 2009).

### Genome assembly and annotation

Mitogenomes from three *Chrysanthemum* accessions were assembled with short read (Illumina) or long-read sequencing (PacBio HiFi) data. Specifically, for the diploid- and tetraploid-*Chrysanthemum*, first, a total of three Gb of reads were randomly extracted from the PacBio HiFi data by using SeqKit v2.1.0 (Shen et al., 2016). Then the published mitogenome of *C. indicum* (NCBI accession number: MH716014.1) was used as a reference to extract mitochondrial reads by using minimap2 with the parameter settings’-cx map-hifi -H’ (Li, 2018). Flye-meta v2.8.3-b1695 (Kolmogorov et al., 2020) was used to assembly the mitogenomes with PacBio HiFi reads, with the master circle conformation mitogenome of *C. indicum* (2x) and the complex mitogenome conformation of *C. indicum* (4x) with multiple connections obtained. Next the reference and *C. indicum* (2x) mitogenomes were mapped to *C. indicum* (4x) to simplify it to a single circle in Bandage (Wick et al., 2015). For *C. indicum* var. *aromaticum* with short read data, a total of five Gb reads were randomly extracted. GSAT was used to assembly the mitogenome with the pipeline ‘graphShort’, then simplified the genome in the same method as above (He et al., 2022). The three single circles were annotated by GeSeq (Tillich et al., 2017)with the reference mitogenome of *C. indicum* (MH716014), and the trans-spliced genes checked manually.

A similar pipeline as mitogenome assembly was used to assemble the plastome of diploid- and tetraploid-*Chrysanthemum* accessions. Given the high copy number of plastome sequences within a cell, one Gb of HiFi reads were randomly extracted for assembly. For *C. indicum* var. *aromaticum* the plastome was extracted from the *de novo* assembly results of GSAT from above. The plastomes were annotated by GeSeq (Tillich et al., 2017; Qu et al., 2019) and PGA software, using the reference plastome of *C. indicum* (NC_020320).

### Synteny fragment analysis

The program Blastn v2.11.0+ with parameter settings ‘-evalue 1e-6’ was used to identify syntenic sequences between the seven accessions from *Chrysanthemum* (Camacho et al., 2009). Syntenic sequences longer than 1 Kb were visualized with NGenomeSyn v1.41 (He et al., 2023).

### Identification of repeat sequences

The repeat types, F (forward), P (palindrome), R (reverse), and C (complement) of dispersed repeat sequences in the seven *Chrysanthemum* accessions organelles were detected using REPuter with parameter settings ‘-c -f -p -r -l 20 -h 3 -best 300’ (Kurtz et al., 2001). The MISA software was used to identify simple sequence repeats (SSRs) with 10, 6, 5, 5, 5, and 5 repeat units set as minimum thresholds for mono-, di-, tri-, tetra-, penta-, and hexa-motifs respectively (Beier et al., 2017).The results were visualized with ggplot2 (Villanueva and Chen, 2019).

### Analysis of plastid-derived sequences in mitogenomes

To determine the plastid-derived sequences within *Chrysanthemum*, the seven mitogenomes were searched against their corresponding plastomes by Blastn v2.11.0+ with parameter settings ‘-evalue 1e-6’. Then the plastid-derived sequences were annotated. The location of plastid-derived sequences in both the mitogenome (destination) and plastome (origin) were visualized with Circos v0.69-9 (Krzywinski et al., 2009). The plastid-derived sequences in mitogenomes were annotated by GeSeq (Tillich et al., 2017)and PGA software, using the reference plastome of *C. indicum* (NC_020320).

### Phylogenetic analysis

To analyze the phylogenetic relationships among Asteraceae, the plastomes and mitogenomes of *C. indicum* (2x), *C. indicum* (4x), *C. indicum* var. *aromaticum*, and 23 other Asteraceae were used, with *Platycodon. grandiflorus* (Campanulaceae) as an outgroup. The CDSs of 80 chloroplast protein-coding genes and 32 mitochondrial protein-coding genes were extracted, concatenated, and aligned using MAFFT v7.490 (Katoh et al., 2002) with poorly aligned sections trimmed with TrimAL v1.4 (Capella-Gutiérrez et al., 2009). These datasets were then used to conduct two separate phylogenetic analysis using IQ-TREE v2.0 with 1000 ultrafast bootstrap replicates to assess branch support based on the auto-selected best-fit model ‘TVM + F + R2’, with FigTree v1.4.3 used for tree visualization (Minh et al., 2020).

All gene pairs alignments were converted into PAML format using EasyCodeML (Yang, 2007; Gao et al., 2019). All the sequences pairs was merge in one file. Molecular divergence times were estimated by placing soft boundaries on the split nodes using records from the timetree database (http://timetree.org/). For divergence time markers we used two nodes, one for the divergence of the outgroup from the Asteraceae, set at 67.7-93.0 Mya, and the other divergence time for the node represented by *Arctium lappa* from the node represented by Chrysanthemum indicum, set at 36.6-45.1 Mya. The MCMCtree module of PAML software was implemented to estimate the divergence time of each node (Dos Reis, 2022). Tracer was used to evaluate whether the results of MCMCtree have converged. The result of divergence time tree results are visualized using ggtree package (Xu et al., 2022).

## Data availability statement

The mitogenome and plastome sequences supporting the conclusions of this article are available in GenBank (https://www.ncbi.nlm.nih.gov/) repository, accession numbers: NC_067879, NC_042378, NC_042756, NC_042406, NC_034354, MH716014, NC_039757, ON053202, OU343227.1, NC_064134, NC_062671, NC_062672, NC_060635, NC_062670, NC_051989, NC_051990, NC_058585, NC_023337, NC_053927, NC_066407, NC_059793, NC_058644, NC_066808, and NC_035958 for mitogenome, MK905238, MH375874, OK128342, MZ127827, NC_060634, MW691204, NC_058915, KX822074, NC_037388, NC_020320, OU343226.1, MK604174, KX063880, NC_007977, MT302568, MT302567, MG696658, NC_066756, DQ383816, NC_066758, NC_067730, NC_050977, NC_031396, and NC_035624 for plastome. The data presented in the study are deposited in the GenBank repository, accession number OQ835564 -OQ835569 and the Genome Sequence Archive (Wang et al., 2017) in the National Genomics Data Center (Partners, 2023), Beijing Institute of Genomics (China National Center for Bioinformation), Chinese Academy of Sciences repository, accession number CRA011215.

## Author contributions

WZ and SZ conceived the study. HH and ZW assembled and annotated the mitogenome and carried out the comparative analysis. LX, QM, and MW analyzed the structural and sequence of plastid genomes. HH, ZW, SZ, LT, and WZ discussed the results. HH, ZW, SZ, and LT wrote the manuscript. WZ and SZ revised the paper. All authors approved the final manuscript.
