# Supplementary material for: Comparative analysis of organellar genomes between diploid and tetraploid Chrysanthemum indicum with its relatives
Source: Front Plant Sci. 2023 Aug 18;14:1228551. doi: 10.3389/fpls.2023.1228551 (PMC10471889; doi:10.3389/fpls.2023.1228551)
Supplement: Supplementary file 1 [file DataSheet_1.docx]

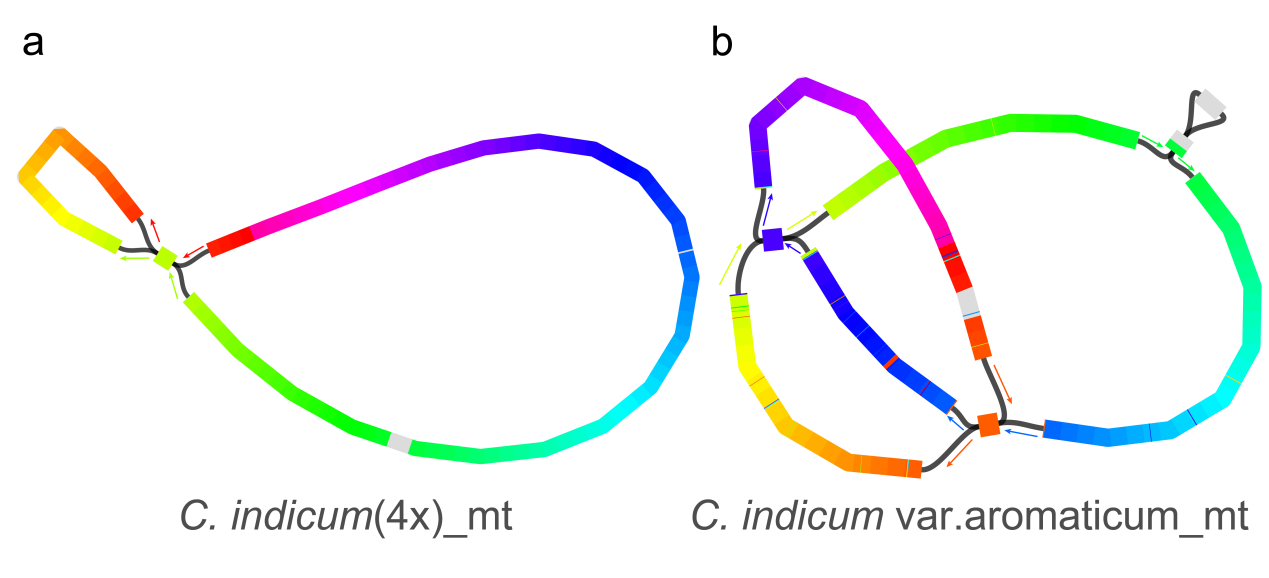


**Fig S1.** The final mitogenome conformation process of . The conformation is from the mitogenome of *C. indicum*(4x) and *C. indicum*. var. aromaticum, the color-coded portion is the mitogenome of *C. indicum*(2x), which were compared to the conformation, from red to pink, representing the continuity of the mitogenome.


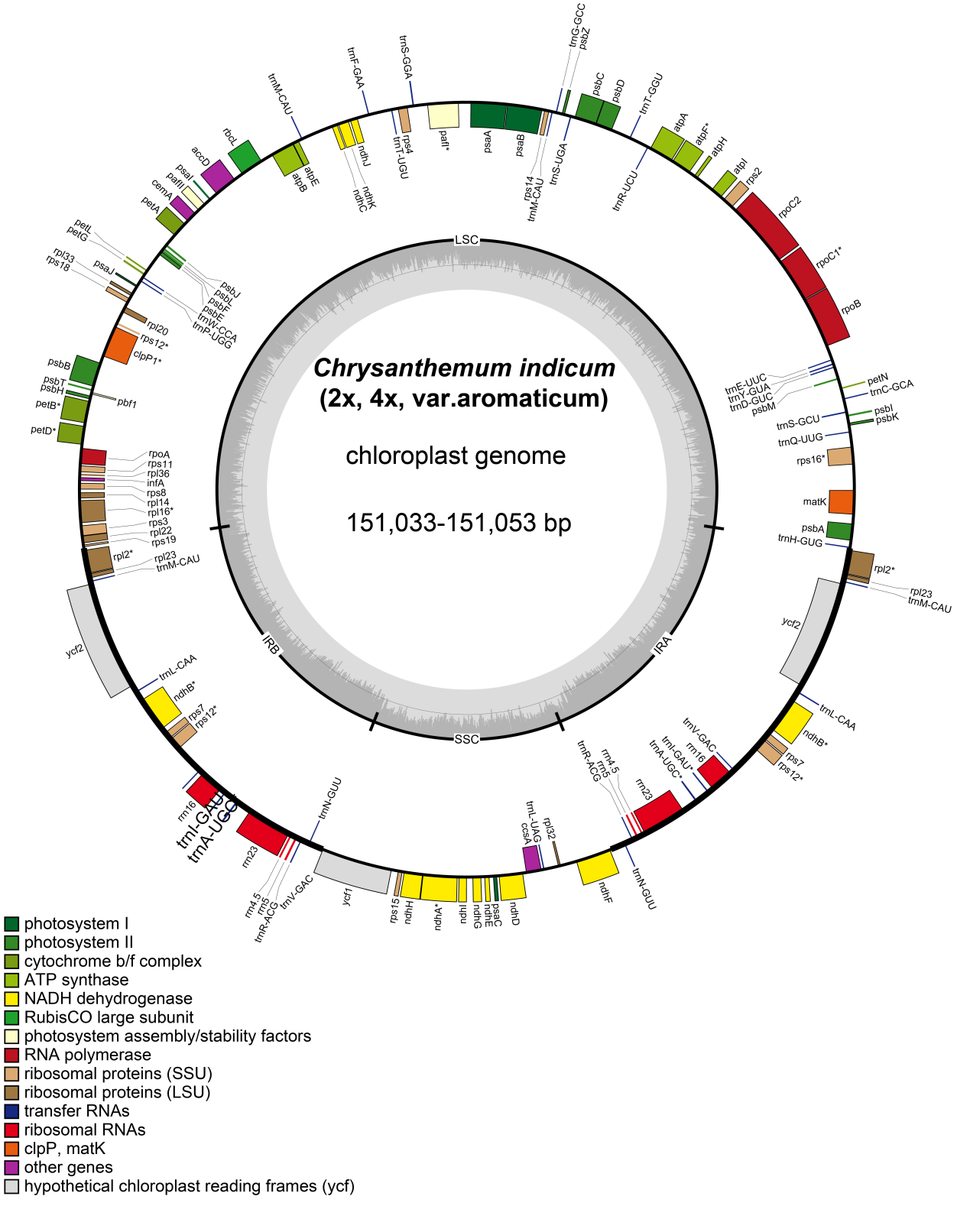


**Fig S2.** Plastome structure of *C. indicum*. Genes mapped outside the outer circle are transcribed counterclockwise, and those inside are transcribed clockwise. Genes are color coded by functional group. The LSC (large single copy region), SSC (small single copy region), and the IRA and IRB (inverted repeats) are indicated on the inner circle along with GC content in dark gray and AT content in lighter gray.


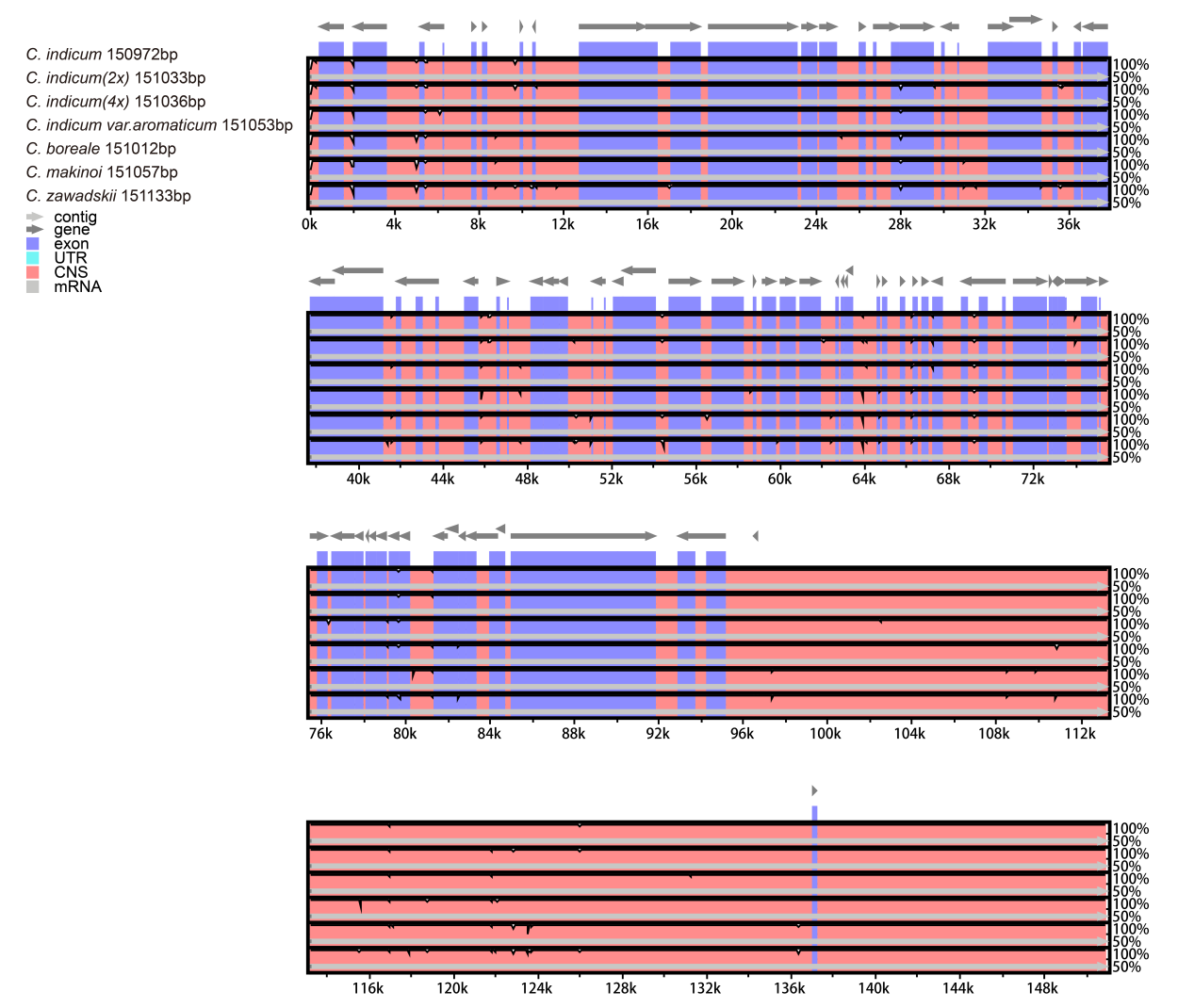


**Fig S3.** Plastome structure of *C. indicum*. Genes mapped outside the outer circle are transcribed counterclockwise, and those inside are transcribed clockwise. Genes are color coded by functional group. The LSC (large single copy region), SSC (small single copy region), and the IRA and IRB (inverted repeats) are indicated on the inner circle along with GC content in dark gray and AT content in lighter gray.


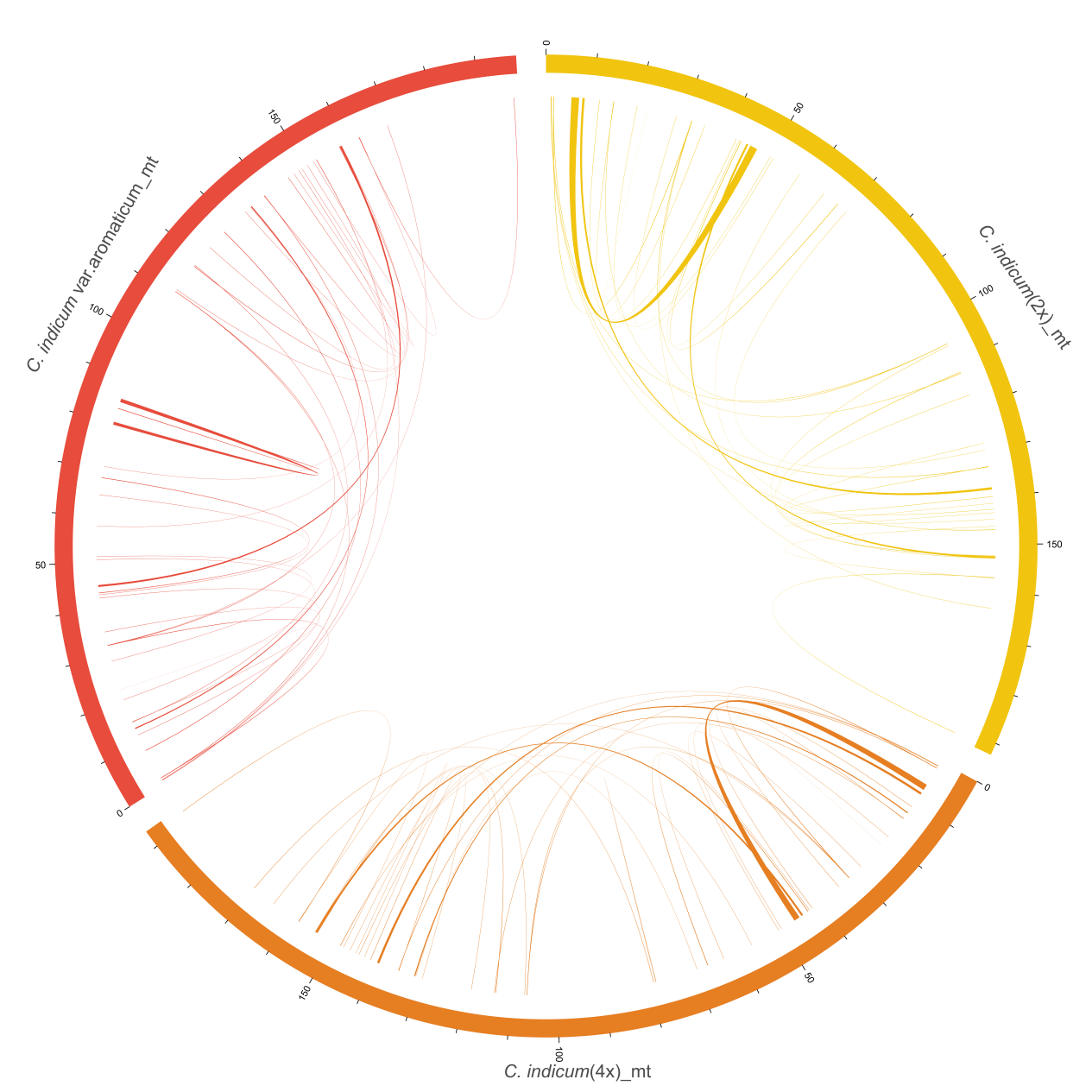


**Fig S4.** Individual internal synteny indicated by all identified homologous regions of *C. indicum*(4x), *C. indicum*(4x) and *C. indicum*. var. aromaticum.


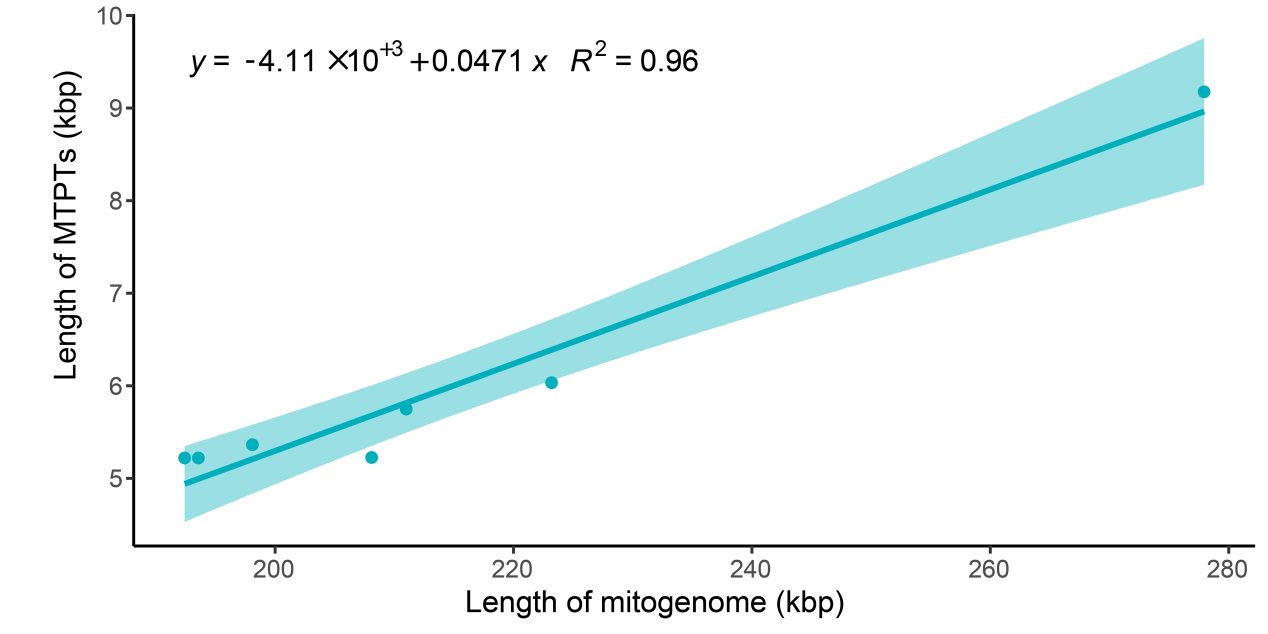


**Fig S5.** Correlation between the total length of MTPTs and the size of the source mitogenome from *Chrysanthemum* genus.


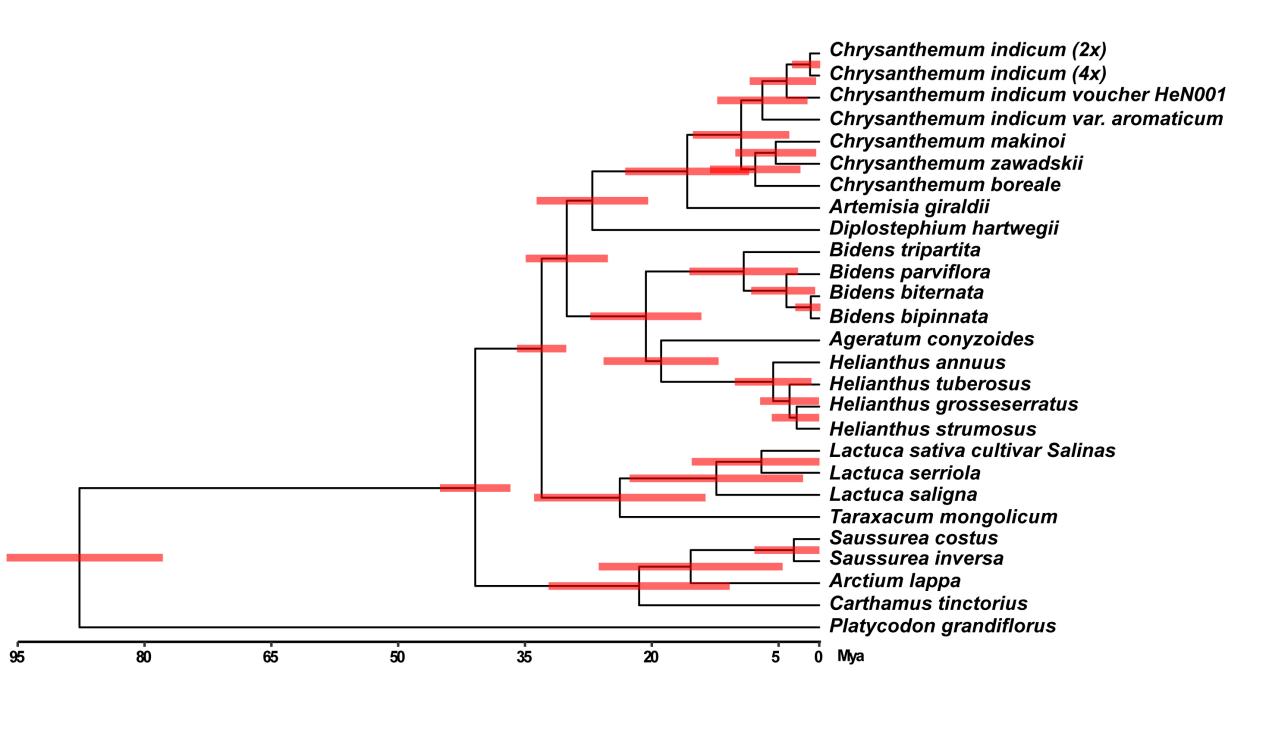


Fig S6. The divergence time among Asteraceae based on mitochondrial genes.


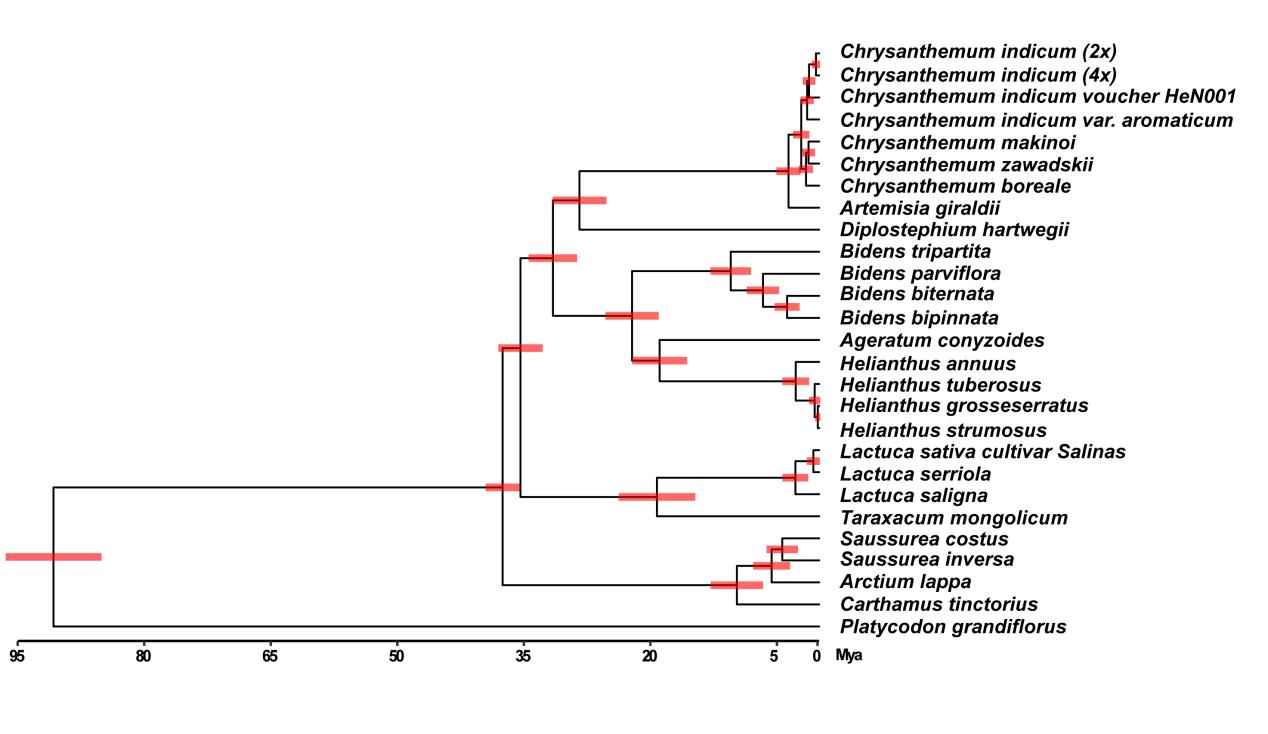


Fig S7. The divergence time among Asteraceae based on chloroplast genes.
